# Supplementary figures and images for: In-depth study of pyroptosis-related genes and immune infiltration in colon cancer
Source: PeerJ. 2024 Oct 29;12:e18374. doi: 10.7717/peerj.18374 (PMC11529595; doi:10.7717/peerj.18374)

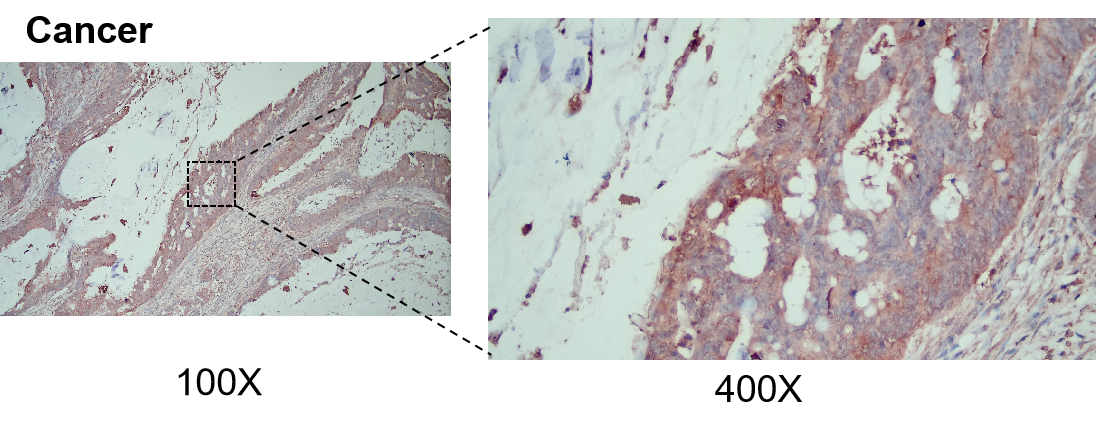

Supplement: Supplemental Information 4 [file peerj-12-18374-s004.zip › RAW DATE/IHC/cancer.tif]

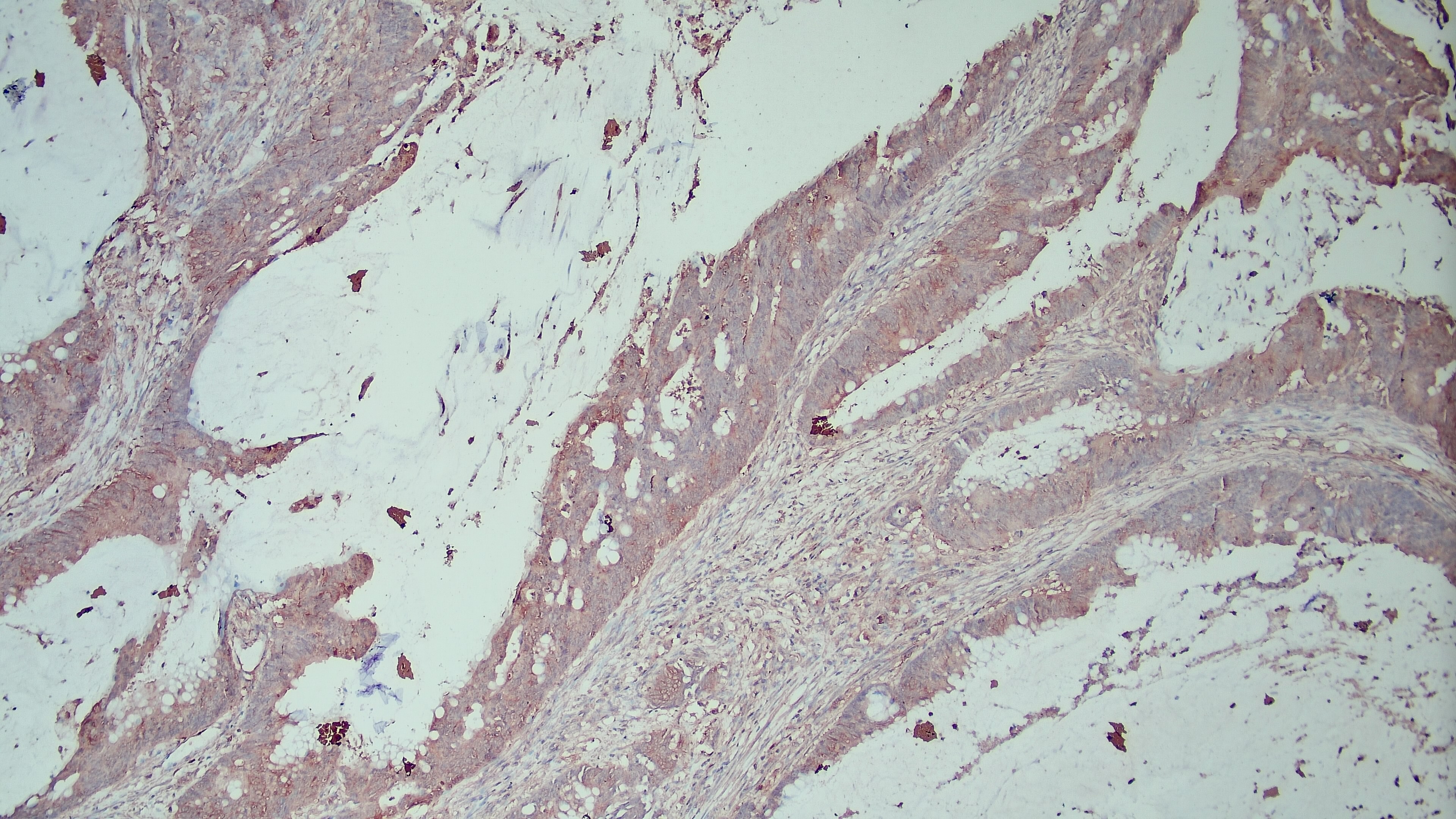

Supplement: Supplemental Information 4 [file peerj-12-18374-s004.zip › RAW DATE/IHC/colon cancer 100X.jpg]

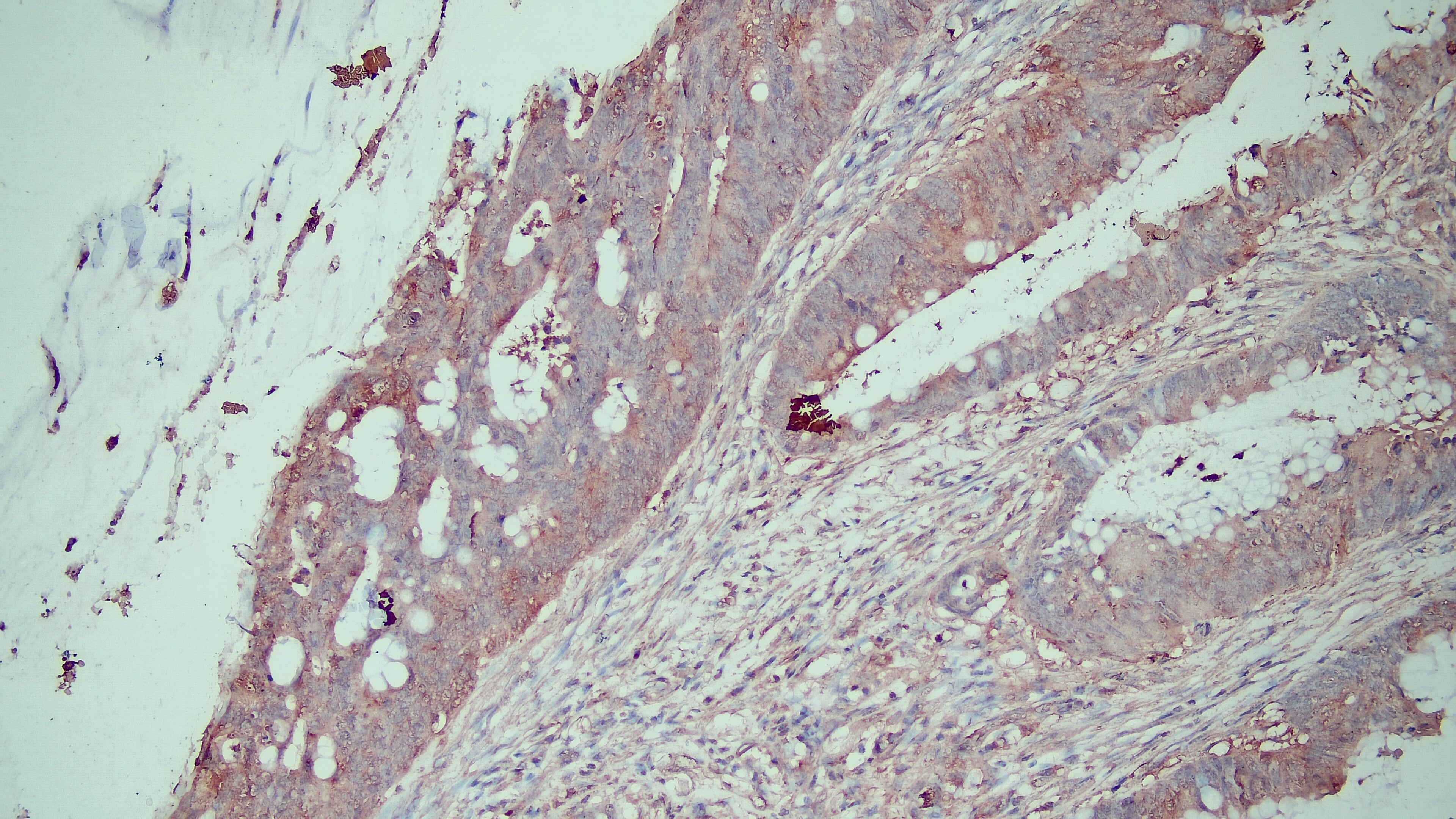

Supplement: Supplemental Information 4 [file peerj-12-18374-s004.zip › RAW DATE/IHC/colon cancer 400X.jpg]

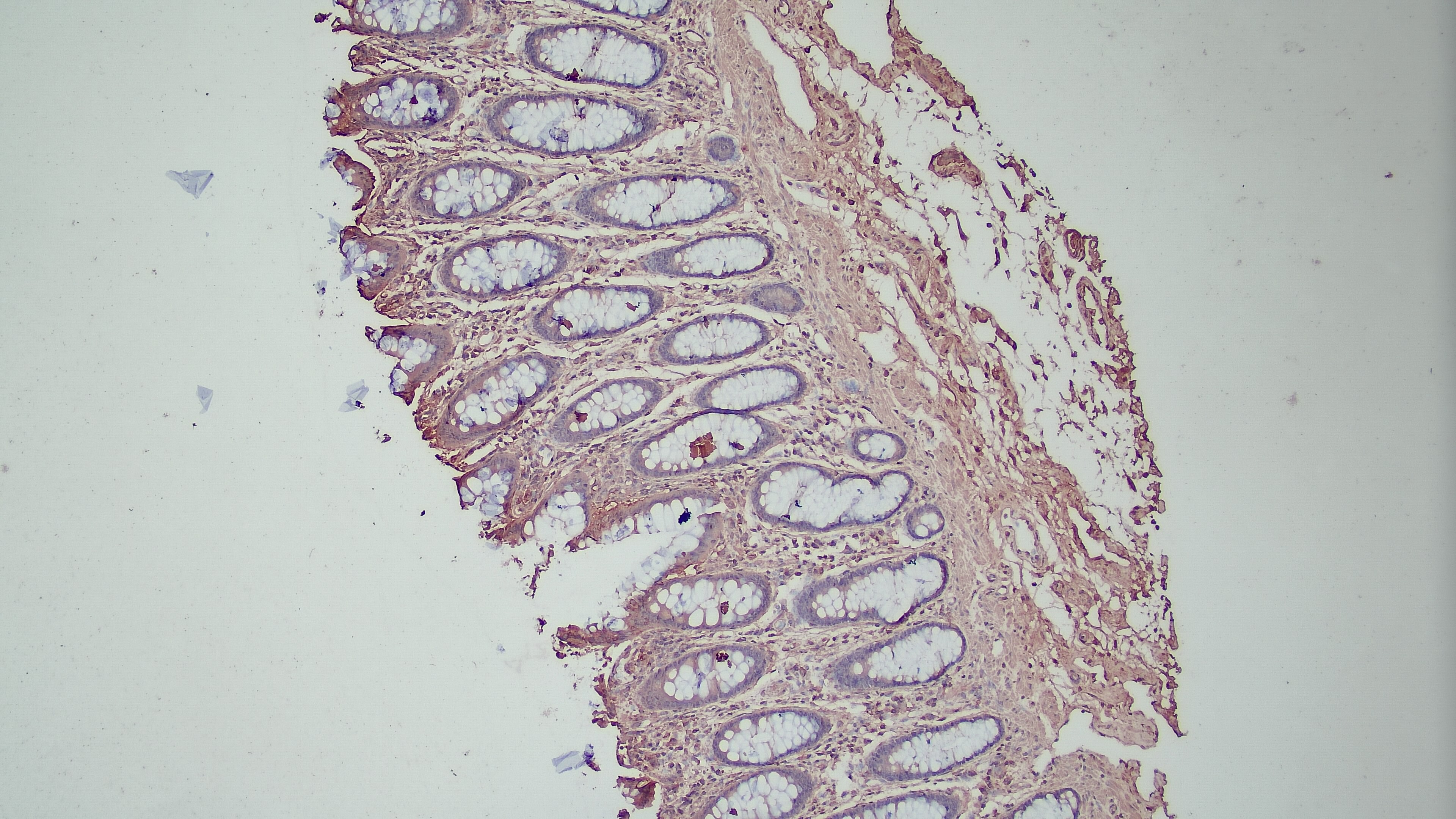

Supplement: Supplemental Information 4 [file peerj-12-18374-s004.zip › RAW DATE/IHC/normal colon tissue 100X.jpg]

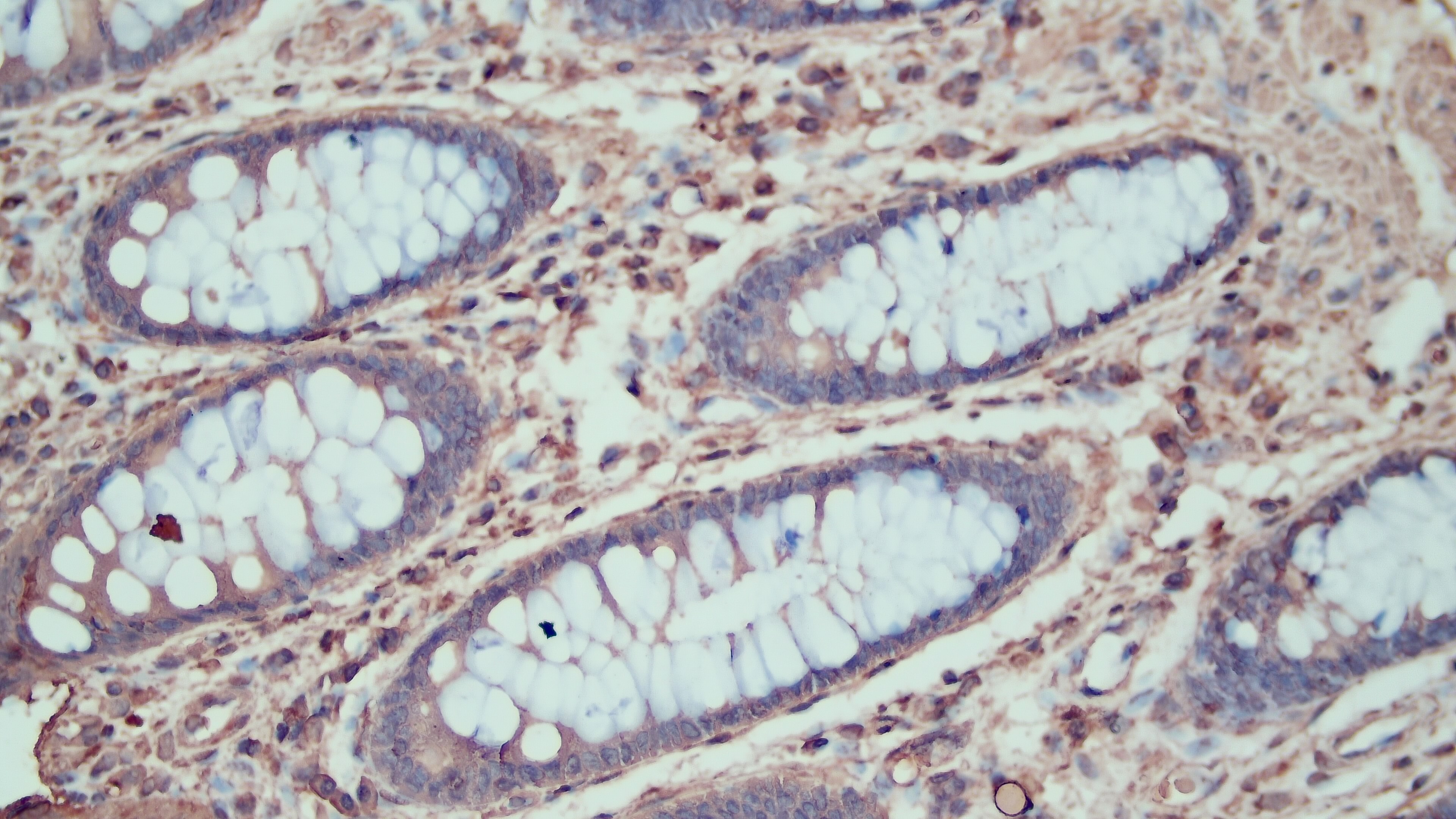

Supplement: Supplemental Information 4 [file peerj-12-18374-s004.zip › RAW DATE/IHC/normal colon tissue 400X.jpg]

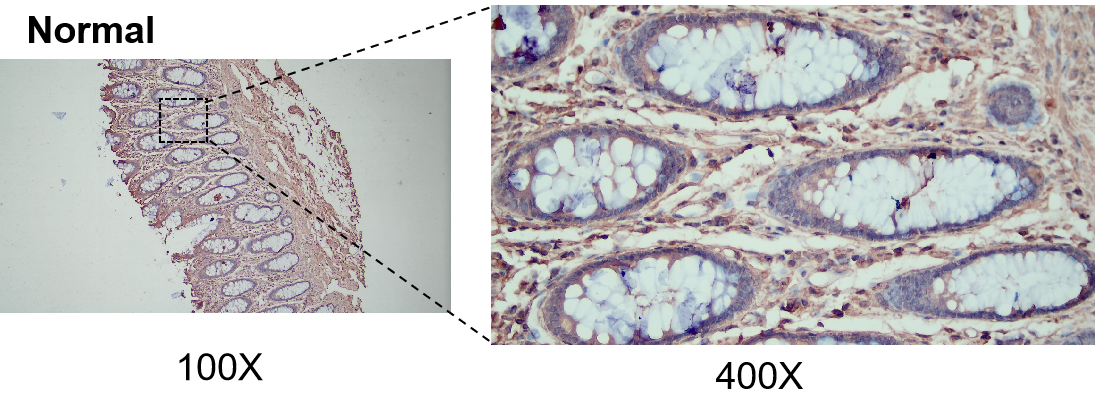

Supplement: Supplemental Information 4 [file peerj-12-18374-s004.zip › RAW DATE/IHC/normal.tif]
