# Supplementary material for: In-depth study of pyroptosis-related genes and immune infiltration in colon cancer
Source: PeerJ. 2024 Oct 29;12:e18374. doi: 10.7717/peerj.18374 (PMC11529595; doi:10.7717/peerj.18374)
Supplement: Supplemental Information 6 [file peerj-12-18374-s006.docx]

**STable 1. GO enrichment analysis**

| **class** | **GO ID** | **Description** | **number** | **pvalue** |
| --- | --- | --- | --- | --- |
| Molecular Function | GO:0030545 | receptor regulator activity | 33 | 1.42E-07 |
| Molecular Function | GO:0048018 | receptor ligand activity | 29 | 1.50E-06 |
| Molecular Function | GO:0008188 | neuropeptide receptor activity | 7 | 5.68E-05 |
| Molecular Function | GO:0005198 | structural molecule activity | 31 | 0.0004087 |
| Molecular Function | GO:0008528 | G protein-coupled peptide receptor activity | 11 | 0.0004296 |
| Molecular Function | GO:0001653 | peptide receptor activity | 11 | 0.0005377 |
| Molecular Function | GO:0005251 | delayed rectifier potassium channel activity | 5 | 0.0006868 |
| Molecular Function | GO:0005249 | voltage-gated potassium channel activity | 8 | 0.0007175 |
| Molecular Function | GO:0004869 | cysteine-type endopeptidase inhibitor activity | 6 | 0.0008989 |
| Molecular Function | GO:0031720 | haptoglobin binding | 3 | 0.0010987 |
| Molecular Function | GO:0070576 | vitamin D 24-hydroxylase activity | 2 | 0.0013996 |
| Molecular Function | GO:0005267 | potassium channel activity | 9 | 0.0016739 |
| Molecular Function | GO:0030547 | receptor inhibitor activity | 5 | 0.0016811 |
| Molecular Function | GO:0001540 | amyloid-beta binding | 7 | 0.0022145 |
| Molecular Function | GO:0042923 | neuropeptide binding | 4 | 0.0022685 |
| Molecular Function | GO:0039706 | co-receptor binding | 3 | 0.0024942 |
| Molecular Function | GO:0015079 | potassium ion transmembrane transporter activity | 10 | 0.0027087 |
| Molecular Function | GO:0004966 | galanin receptor activity | 2 | 0.0027589 |
| Molecular Function | GO:0045499 | chemorepellent activity | 4 | 0.0030023 |
| Molecular Function | GO:0015267 | channel activity | 20 | 0.0032301 |
| Cellular Component | GO:0005576 | extracellular region | 143 | 2.12E-08 |
| Cellular Component | GO:0044421 | extracellular region part | 118 | 7.49E-07 |
| Cellular Component | GO:0005615 | extracellular space | 110 | 2.50E-06 |
| Cellular Component | GO:0031226 | intrinsic component of plasma membrane | 62 | 5.68E-05 |
| Cellular Component | GO:0005887 | integral component of plasma membrane | 59 | 8.99E-05 |
| Cellular Component | GO:0032809 | neuronal cell body membrane | 5 | 0.0002609 |
| Cellular Component | GO:0034705 | potassium channel complex | 9 | 0.0003122 |
| Cellular Component | GO:0044298 | cell body membrane | 5 | 0.0004365 |
| Cellular Component | GO:0008076 | voltage-gated potassium channel complex | 8 | 0.0007765 |
| Cellular Component | GO:0044445 | cytosolic part | 14 | 0.0009771 |
| Cellular Component | GO:0044297 | cell body | 25 | 0.0013875 |
| Cellular Component | GO:0030424 | axon | 27 | 0.0013981 |
| Cellular Component | GO:0031838 | haptoglobin-hemoglobin complex | 3 | 0.0014893 |
| Cellular Component | GO:1903561 | extracellular vesicle | 66 | 0.0020871 |
| Cellular Component | GO:0043230 | extracellular organelle | 66 | 0.0021355 |
| Cellular Component | GO:0005833 | hemoglobin complex | 3 | 0.0024991 |
| Cellular Component | GO:0070062 | extracellular exosome | 65 | 0.0025738 |
| Cellular Component | GO:0005791 | rough endoplasmic reticulum | 7 | 0.0030997 |
| Cellular Component | GO:0022627 | cytosolic small ribosomal subunit | 5 | 0.003158 |
| Cellular Component | GO:0034703 | cation channel complex | 12 | 0.0041324 |
| Biological Process | GO:0032501 | multicellular organismal process | 220 | 1.79E-09 |
| Biological Process | GO:0032502 | developmental process | 185 | 6.71E-08 |
| Biological Process | GO:0048513 | animal organ development | 118 | 2.85E-07 |
| Biological Process | GO:0007275 | multicellular organism development | 161 | 5.30E-07 |
| Biological Process | GO:0048856 | anatomical structure development | 169 | 2.81E-06 |
| Biological Process | GO:0009605 | response to external stimulus | 90 | 3.79E-06 |
| Biological Process | GO:0048869 | cellular developmental process | 133 | 4.74E-06 |
| Biological Process | GO:0048731 | system development | 143 | 7.09E-06 |
| Biological Process | GO:0030154 | cell differentiation | 128 | 7.10E-06 |
| Biological Process | GO:0010817 | regulation of hormone levels | 29 | 9.02E-06 |
| Biological Process | GO:0051093 | negative regulation of developmental process | 42 | 1.06E-05 |
| Biological Process | GO:0051239 | regulation of multicellular organismal process | 101 | 1.71E-05 |
| Biological Process | GO:0045653 | negative regulation of megakaryocyte differentiation | 5 | 2.73E-05 |
| Biological Process | GO:0045596 | negative regulation of cell differentiation | 33 | 3.72E-05 |
| Biological Process | GO:0050896 | response to stimulus | 233 | 4.44E-05 |
| Biological Process | GO:0042572 | retinol metabolic process | 7 | 6.01E-05 |
| Biological Process | GO:0051241 | negative regulation of multicellular organismal process | 47 | 6.02E-05 |
| Biological Process | GO:0048239 | negative regulation of DNA recombination at telomere | 6 | 6.78E-05 |
| Biological Process | GO:0072695 | regulation of DNA recombination at telomere | 6 | 6.78E-05 |
| Biological Process | GO:0010739 | positive regulation of protein kinase A signaling | 4 | 8.32E-05 |
