# Supplementary material for: In-depth study of pyroptosis-related genes and immune infiltration in colon cancer
Source: PeerJ. 2024 Oct 29;12:e18374. doi: 10.7717/peerj.18374 (PMC11529595; doi:10.7717/peerj.18374)
Supplement: Supplemental Information 7 [file peerj-12-18374-s007.docx]

**STable 2. KEGG pathway enrichment analysis**

| **KEGG ID** | **Pathway** | **Number** | **Pvalue** |
| --- | --- | --- | --- |
| ko04080 | Neuroactive ligand-receptor interaction | 21 | 2.65E-05 |
| ko04978 | Mineral absorption | 7 | 3.46E-04 |
| ko04970 | Salivary secretion | 8 | 9.44E-04 |
| ko00830 | Retinol metabolism | 6 | 3.61E-03 |
| ko04261 | Adrenergic signaling in cardiomyocytes | 9 | 6.08E-03 |
| ko03320 | PPAR signaling pathway | 6 | 7.99E-03 |
| ko05034 | Alcoholism | 10 | 1.05E-02 |
| ko00140 | Steroid hormone biosynthesis | 5 | 1.36E-02 |
| ko04610 | Complement and coagulation cascades | 6 | 1.46E-02 |
| ko04977 | Vitamin digestion and absorption | 3 | 1.67E-02 |
| ko04723 | Retrograde endocannabinoid signaling | 8 | 2.04E-02 |
| ko05322 | Systemic lupus erythematosus | 10 | 2.08E-02 |
| ko04976 | Bile secretion | 5 | 2.28E-02 |
| ko04916 | Melanogenesis | 6 | 2.42E-02 |
| ko04310 | Wnt signaling pathway | 8 | 3.01E-02 |
| ko04728 | Dopaminergic synapse | 7 | 3.17E-02 |
| ko04913 | Ovarian Steroidogenesis | 4 | 3.19E-02 |
| ko00512 | Mucin type O-glycan biosynthesis | 3 | 3.22E-02 |
| ko05020 | Prion diseases | 3 | 4.05E-02 |
| ko04911 | Insulin secretion | 5 | 4.90E-02 |
